# Magnetic Excitations of Isolated and Interconnected Complexes on a Superconductor

**DOI:** 10.1021/acs.nanolett.5c04828

**Published:** 2025-11-11

**Authors:** Xiangzhi Meng, Jenny Möller, Martin Irizar, Daniel Sánchez-Portal, Aran Garcia-Lekue, Alexander Weismann, Rainer Herges, Richard Berndt

**Affiliations:** † Institut für Experimentelle und Angewandte Physik, 9179 Christian-Albrechts-Universität, 24098 Kiel, Germany; ‡ Otto-Diels-Institut für Organische Chemie, Christian-Albrechts-Universität, 24098 Kiel, Germany; ¶ 226245 Donostia International Physics Center (DIPC), 20018 Donostia-San Sebastián, Spain; § Department of Polymers and Advanced Materials: Physics, Chemistry and Technology, Faculty of Chemistry, University of the Basque Country UPV/EHU, 20018 San Sebastián, Spain; ∥ Centro de Física de Materiales CSIC-UPV/EHU, 20018 Donostia-San Sebastián, Spain; ⊥ Ikerbasque, Basque Foundation for Science, 48013 Bilbao, Spain

**Keywords:** spin-flip excitation, YSR, scanning tunneling microscopy

## Abstract

The magnetic complex Fe­(II) porphyrin was studied on superconducting Pb(100). Low-temperature scanning tunneling microscopy was performed on isolated molecules and molecular networks prepared via Ullmann coupling. Isolated molecules adopt two distinct adsorption configurations with different magnetic excitations, namely, Yu-Shiba-Rusinov states and spin excitations. They may be understood in terms of their different magnetic anisotropies and exchange couplings with the substrate, as illustrated by the density functional theory calculations. In networks, strong intermolecular interactions lead to diverse structures with distinct magnetic and electronic properties. This work demonstrates a degree of control over the magnetic quantum states of a molecular complex on a superconductor.

## Graphical abstract

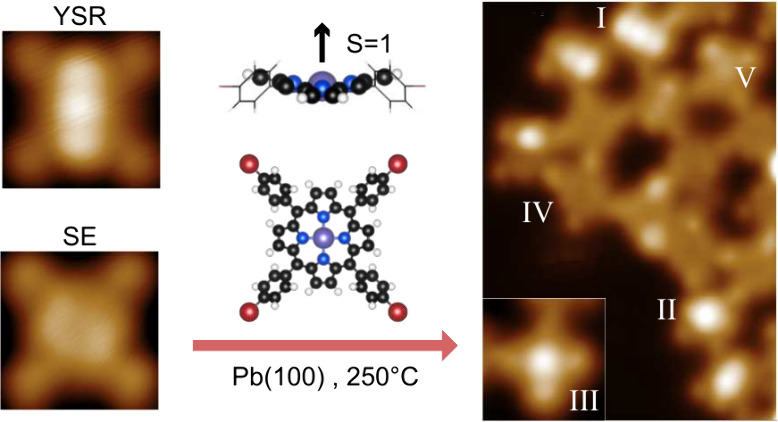

Magnetic atoms and molecules residing on superconducting surfaces have attracted attention for their potential to host topological phases and their possible role as building blocks in quantum computing devices.^1–4^ The interaction with the environment can significantly impact the magnetic characteristics of the adsorbates and may be used to tailor magnetic properties for specific applications.^5–7^

On a superconducting surface, a localized spin may undergo exchange scattering with the substrate quasiparticles, leading to the formation of bound states known as Yu-Shiba-Rusinov (YSR) states.^8–10^ In scanning tunneling spectroscopy (STS), YSR states appear as pair(s) of resonances located symmetrically around the Fermi energy *E*_*F*_ within the superconducting energy gap. The position of these resonances is governed by the strength, *J*, of the exchange scattering. Variations in *J* can lead to a quantum phase transition, i*.*e*.*, a change of the ground state between a free-spin state and a screened-spin state.^11–13^ Within the superconducting gap, multiple pairs of YSR resonances can arise from distinct mechanisms such as different scattering channels associated with the internal orbital structure,^14,15^ energy-level splitting due to intrinsic magnetic anisotropy,^16,17^ and coupling to molecular vibrations.^18^ In addition to in-gap states, spin-flip excitations (SE) can be detected as resonances outside the gap.^5,6,19,20^ They appear symmetrically around the Fermi level, and their energies are determined by the axial (*D*) and transverse (*E*) anisotropy parameters.

For magnetic impurities with spin *S* > 1/2, both YSR and SE can be observed.^6,21^ Previous studies have investigated molecules either as isolated adsorbates on surfaces or within self-assembled monolayers, where the magnetic anisotropy of the impurity and the exchange coupling with the substrate are restricted to a narrow range by available adsorption configurations. To explore a broader parameter space, on-surface reactions can be utilized, as they promote intermolecular interactions and enable a wider variety of molecular adsorption geometries.

We investigated the magnetic complex Fe­(II)-5,10,15,20-tetrakis­(4′-bromophenyl)­porphyrin (FeTBrPP) on a Pb(100) surface using low-temperature scanning tunneling microscopes (STM). The Br substitution enables Ullmann coupling, leading to the formation of a 2D network interconnected by C–C single bonds. We first present data from pristine molecules prior to the coupling reaction. Isolated adsorbed molecules adopt two distinct configurations, exhibiting either YSR states or SE. By modification of the adsorption site, these excitations may be interconverted. We carried out numerical renormalization group (NRG) calculations^22–25^ to understand the dependence of the YSR resonances on *D* and *J*. Density functional theory (DFT) calculations reveal that both configurations possess spin *S* = 1. Molecules within Ullmann-coupled networks display new topographic features. Moreover, the magnetic anisotropy of the molecules exhibiting SE varies by an order of magnitude (*D* = 0.9–11.6 meV). For molecules displaying YSR states, different quantum phases are identified as *J* is varied. Finally, modification of the tip–molecule distance tunes both *D* and *J*.

Upon adsorption on a Pb(100) surface held at room temperature, FeTBrPP molecules self-assemble into molecular islands (Figure 1a). Similar to previously reported porphyrins,^26–30^ most FeTBrPP molecules adopt a saddle shape, with two pyrrole groups pointing upward and the other two pointing downward. The axis along the diagonal Br atoms of the saddle-shaped molecule (black dashed line in Figure 1a) is aligned with the ⟨011⟩ direction of the surface. The center of the saddle molecules is located on the top sites of the substrate lattice (Figure 1b). We denote these molecules as T-type.

**Figure 1:**
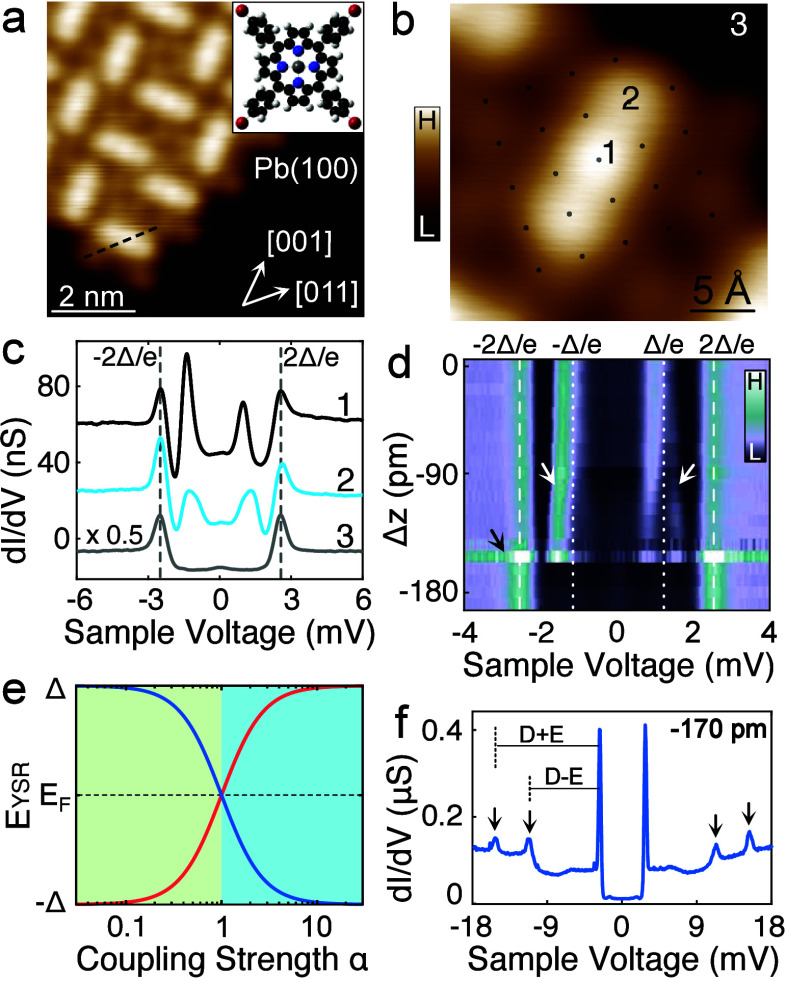
FeTBrPP molecule layer on Pb(100). (a) STM topograph (−60 mV, 50 pA) of a molecular island on Pb(100). The inset shows the molecular structure. Black, blue, white, red, and gray spheres represent C, N, H, Br, and Fe atoms, respectively. (b) Zoom-in STM image (−60 mV, 30 pA) of an FeTBrPP molecule in an island. The black dots indicate the positions of Pb surface atoms underneath. (c) d*I*/d*V* spectra recorded at the molecule center (1), pyrrole (2), and surface (3) with tip height set at −6 mV and 100 pA. (d) Color map of the d*I*/d*V* spectra recorded at varying tip–sample distances. Tip height at 0 pm is set at −6 mV and 100 pA. The dashed lines at ±2Δ indicate the superconducting coherence peaks of the surface. The dotted lines mark ±Δ. Thermal replicas of the YSR resonances appear in the range from −Δ to Δ. (e) Model YSR energy (blue) as a function of coupling strength α. The YSR resonance at the opposite polarity is colored red. The green and cyan regions represent two different quantum phases, where the ground state is a free and screened spin state, respectively. (f) d*I*/d*V* spectrum of a wider bias range at a tip height of −170 pm.

We performed d*I*/d*V* spectroscopy using a superconducting lead tip to achieve an energy resolution beyond the thermal limit.^12,13,31–33^ The measured d*I*/d*V* spectra are a convolution of the densities of states (DOS) of the tip and substrate, which shifts all spectral features of the substrate by Δ/*e*, where Δ is the superconducting energy gap of the tip. The coherence peaks of the superconducting surface are thus observed at ±2Δ/*e*. A pair of subgap YSR states is observed at both the Fe center and the pyrrole groups, with the latter exhibiting reduced intensity (Figure 1c). The electron- and hole-like YSR excitations at positive and negative sample voltages, respectively, exhibit different intensities. This asymmetry arises from additional nonmagnetic potential scattering between the magnetic impurities and its environment.^13,21^

The YSR energy as a function of the exchange coupling can be described by^34^

1 $$E_{\mathrm{YSR}}=\Delta \frac{1-\alpha^{2}}{1+\alpha^{2}}$$

where Δ is the superconducting order parameter of the sample, and α = *πρJS*_imp_. ρ is the DOS of the sample in the normal state at the Fermi level, and *S*_imp_ is the impurity spin. Equation 1 is depicted by the blue curve in Figure 1e. In the green region, where a free-spin ground state prevails, the YSR resonances shift toward the gap edge as *J* decreases. In contrast, in the cyan region, corresponding to a screened-spin ground state, these resonances shift toward the Fermi level with decreasing *J*.

As the tip approaches the molecule center, the YSR peaks shift toward the gap edge (white arrows in Figure 1d). Meanwhile, the thermally induced replicas of the resonances, located between −Δ/*e* and Δ/*e*, shift toward the Fermi level. This phenomenon can be explained by the attractive force from the tip,^6^ which lifts up the molecule from the substrate and reduces the exchange coupling *J*. The shift toward the gap edge indicated that the ground (excited) state is a free (screened) spin state (green region in Figure 1e). With a further approach, the YSR peaks disappear and SE emerges (Figure 1f). We observed strong spectral noise (black arrow in Figure 1d) in the tip height range during the transition from the YSR to SE. The noise may reflect an instability of the molecular structure. It is worth noting that the YSR states reappear when the tip is lifted up again. From the resonance position (−1.35 mV) at *Δz* = 0 pm, the corresponding exchange coupling is determined to be *ρJ* = 0.3.

Within the molecular layer, a minority of molecules exhibit a different topograph while retaining the same adsorption sites as the saddle molecules (Figure S1). The d*I*/d*V* spectra of these molecules also show SE, suggesting that the molecular structure determines the spin-related excitations.

To vary the adsorption sites and orientations, we laterally moved the molecules using an STM tip. As described below, this manipulation can also interconvert the YSR states and SE.

Figure 2a shows an isolated T-type molecule that had been pulled away from an island onto the pristine surface. Its image contrast and presumably its adsorption configuration are identical to the molecules within islands. Figure 2b shows DFT-optimized geometry. The YSR peaks (Figure 2c) exhibit the same asymmetry and peak positions (stronger intensity at −1.37 mV) as those observed in islands. Spectroscopy over a wide range (Figure 2d) reveals a broad feature between −400 and −50 mV, similar to that observed for FeTPP molecules on Pb(111).^21^ This feature is less pronounced at the down pyrrole site (tip position 3). Akin to the saddle-shaped molecules within islands, the T molecule exhibits no SE.

**Figure 2:**
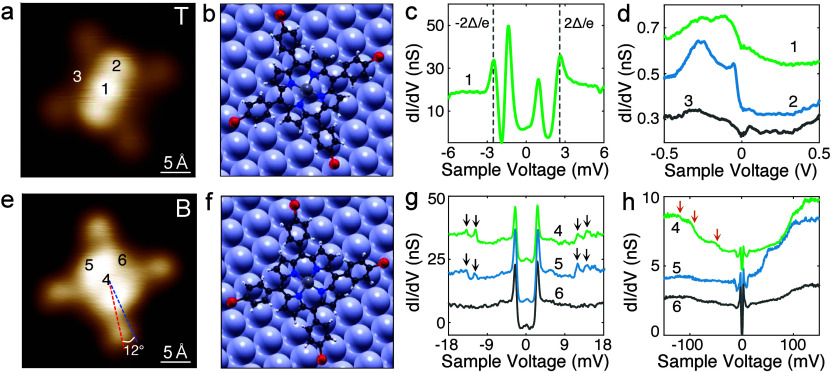
Isolated FeTBrPP molecules at the top (T) and bridge (B) sites of Pb(100). (a, e) STM images (−60 mV, 50 pA) of isolated T-type (a) and B-type (e) molecules. (b, f) Model structures of T-type (b) and B-type (f) molecules optimized via DFT calculations. The black, blue, white, red, gray, and light blue spheres represent C, N, H, Br, Fe, and Pb, respectively. (c, g) d*I*/d*V* spectra recorded above the molecules with tip height set at −6 mV, 100 pA (c) and −30 mV, 250 pA (g). (d, h) Wide range d*I*/d*V* spectra measured at the set points of −500 mV, 150 pA (d) and −200 mV, 500 pA (h).

Manipulation can also induce a different molecular configuration and electronic state (Figure 2e). In this configuration, the molecule appears flatter, with a height difference of ≈10 pm between the neighboring pyrrole groups, compared to >50 pm for T-type molecules (Figure S2). The axis along the diagonal Br atoms (red dashed line in Figure 2e) forms an angle of ≈12° with respect to the ⟨011⟩ direction of the surface (blue dashed line). Further analysis shows that the molecule is centered at a bridge site (Figure 2f). We thus designate it as a B-type molecule. Instead of in-gap YSR states, two pairs of resonances appear outside the superconducting gap, symmetrically located at ±11.8 mV and ±13.8 mV (Figure 2g), which we assign to SE.^5,19^ In a spin system of *S* = 1, easy-plane anisotropy (*D* > 0) with finite transverse anisotropy *E* gives rise to two possible excitations with equal transition probabilities,^5^ leading to two pair of resonances outside the superconducting gap as shown in Figure 1f and Figure 2g. The separations between the SE and coherence peaks are *D* + *E* and *D* – *E* (Figure 1f), respectively. As *E* decreases, the SE peaks gradually approach each other and merge into a single peak when *E* = 0. The derived axial (transverse) anisotropy parameter *D* (*E*) of the isolated B-type molecule is 10.3 meV (1 meV), assuming a molecular spin of *S* = 1, which is further confirmed by the results of DFT calculations. The values of *D* and *E* obtained here are close to previously reported values of Fe porphyrins.^5,35^ The peak intensity nearly vanishes above the lower pyrrole group (position 3), indicating that the SE is strongly position-dependent. On a wider voltage range (Figure 2h), step-like structures are observed at ±52, ±101, and ±123 mV. The conductance step heights are large and exhibit significant dependencies on the bias polarity and the tip position, suggesting that they likely reflect orbital excitations.^36,37^

The transition from the YSR to SE can be attributed to a reduction in the exchange coupling and a substantial increase in the magnetic anisotropy. As the exchange coupling decreases, the YSR resonances gradually shift toward the superconducting gap edge (Figure 1e and Figure 3b). For a fixed exchange coupling, an increase in the magnetic anisotropy induces a similar shift of the YSR resonances (Figure 3a). In particular, a large easy-plane uniaxial anisotropy (*D* > 0) can strongly suppress, or even completely quench, the YSR states.^38^ The absence of in-gap states and the symmetric coherence peaks on the B-type molecule indicate that the exchange coupling with the surface is negligible.

**Figure 3:**
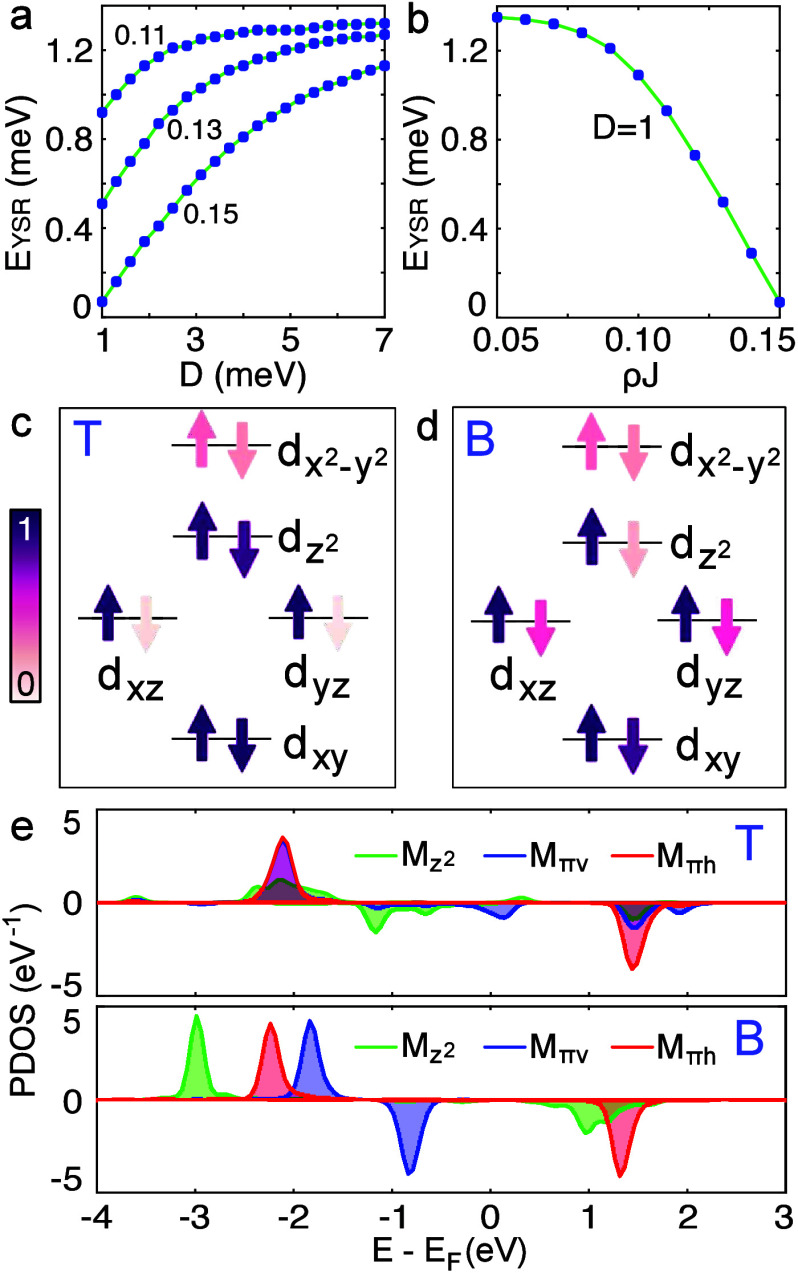
Theoretical results from NRG (a, b) and DFT (c–e) calculations. (a) Energy of YSR resonances as a function of *D* for fixed values of *ρJ* = 0.11, 0.13, and 0.15. (b) Energy of YSR resonances as a function of *ρJ* for *D* = 1 meV. Δ was set to 1.3 meV. (c, d) Schemes of the occupation of the Fe 3*d* levels. (e) Spin-polarized DOS projected on molecular orbitals *M*_*z* ^2^_, *M*_*πv*_, and *M*_*πh*_, which show similar characters as Fe *d*_*z* ^2^_, *d*_*xz*_, and *d*_*yz*_ orbitals.

The experiments show a significant change in the spin-related signal and the electronic structure (Figure S3) between the T- and B-type configurations. To further analyze this effect, we performed DFT calculations. Figure 3c and d present the occupation of the Fe3*d* orbitals. Table 1 summarizes the calculated magnetic moments. Both molecules exhibit a total magnetic moment of approximately 2 μ_*B*_, indicating a consistent spin state of *S* = 1 for the isolated molecules. For both configurations, the *d*_*x* ^2^–*y* ^2^_ orbital is nearly empty, while the *d*_*xy*_ orbital is fully occupied (Figure 3c and d). The primary difference between the T- and B-type molecules arises from the *d*_*z* ^2^_ and *d*_π_ orbitals. For the T-type molecule, the magnetic moment of the *d*_*z* ^2^_ orbital is minimal (0.06 μ_*B*_). The net spin is thus determined by the *d*_*xz*_ and *d*_*yz*_ orbitals. In contrast, the minority *d*_*z* ^2^_ orbital is more occupied in the B-type molecule, at the expense of the *d*_*xz*_ and *d*_*yz*_ orbitals. This results in a substantial increase in the magnetic moment of the *d*_*z* ^2^_ orbital, accompanied by a notable decrease in the moments of the *d*_*xz*_ and *d*_*yz*_ orbitals.

**Table 1:** Calculated Magnetic Moments of the 3*d* Orbitals in Units of *μ*_*B*_

	Total	*d*_*z* ^2^_	*d*_*xz*_	*d*_*yz*_	*d*_*x* ^2^–*y* ^2^_	*d*_*xy*_
T	1.98	0.07	0.89	0.92	0.09	0.01
B	1.99	0.76	0.51	0.49	0.09	0.14

The magnetic anisotropy is governed by the occupation and energy levels of the 3*d* orbitals,^39^ which can be tuned via the tip–molecule distance^5,40^ or by altering the adsorption sites.^29^ Comparison of the orbital occupations for the T- and B-type molecules indicates that the axial anisotropy increases as the occupation of the minority *d*_π_ (*d*_*z* ^2^_) orbital increases (decreases), consistent with previous observations for the same molecule on Au(111).^29^ The pronounced anisotropy observed on B-type molecules may also be linked to their larger structural deformation: the N atoms in the upper and lower pyrrole groups are vertically displaced by +12 and −6 pm, respectively, relative to the Fe atom. For the T-type molecule, the corresponding displacements are slightly smaller, +7 and −5 pm. It may be worth noting that the stronger deformation of the B molecules does not contradict the fairly planar topograph measured with STM because the image contrast primarily reflects the electronic excitations.

Relaxed structural models reveal that the T-type molecule is closer to the surface (341 pm) than the B molecule (359 pm), implying a larger exchange coupling for the T-type molecule. Consistently, Figure 3e indicates stronger hybridization with the substrate, as evidenced by the broader DOS of the T-type molecule compared to that of the B-type. The d*I*/d*V* spectrum recorded on the T-type molecule under a magnetic field displays a Frota line shape,^41^ arising from the Kondo effect (Figure S4). The extracted Kondo temperature, *T*_*K*_ = 5 K, gives *k*_*B*_ *T*_*K*_ < Δ, indicating that the ground state involved in the YSR excitation is a free-spin state.

The above results demonstrate that variations in the adsorption structure lead to significant changes in both the magnetic anisotropy and the exchange coupling with the surface. In the following, Ullmann coupling is employed to generate additional adsorption configurations, resulting in a wider range of magnetic anisotropies and exchange couplings.

Ullmann coupling, i*.*e*.*, the homocoupling of aryl halides, is an on-surface synthesis method used to form C–C bonds between aromatic units on coinage metal surfaces.^42–48^ We found that Ullmann coupling can also occur on the Pb(100) surface. Figure 4a shows a topograph recorded after a Ullmann coupling reaction had been induced by annealing at temperatures up to 540 K (a large-scale overview is shown in Figure S6). Various molecular configurations were obtained and are categorized into different species, labeled I–V. Molecule I shows the same saddle structure as the isolated T-type molecules. The ridge along the [up]­pyrrole-Fe-[up]­pyrrole axis exhibits a similar height ranging from 200 to 240 pm. Figures 4b–e shows detailed topographs of species II–V. The Fe centers in molecules II and III appear higher than those in molecules IV and V. Figure 4f shows line profiles of species II–V along the horizontal axis (dashed lines in Figure 4b–e). The center of molecule II is higher and broader than that of molecule III. The molecular heights of both molecules IV and V are less than 200 pm. Among these species, molecule IV, with its flatter center, is the most prevalent species on the surface.

**Figure 4:**
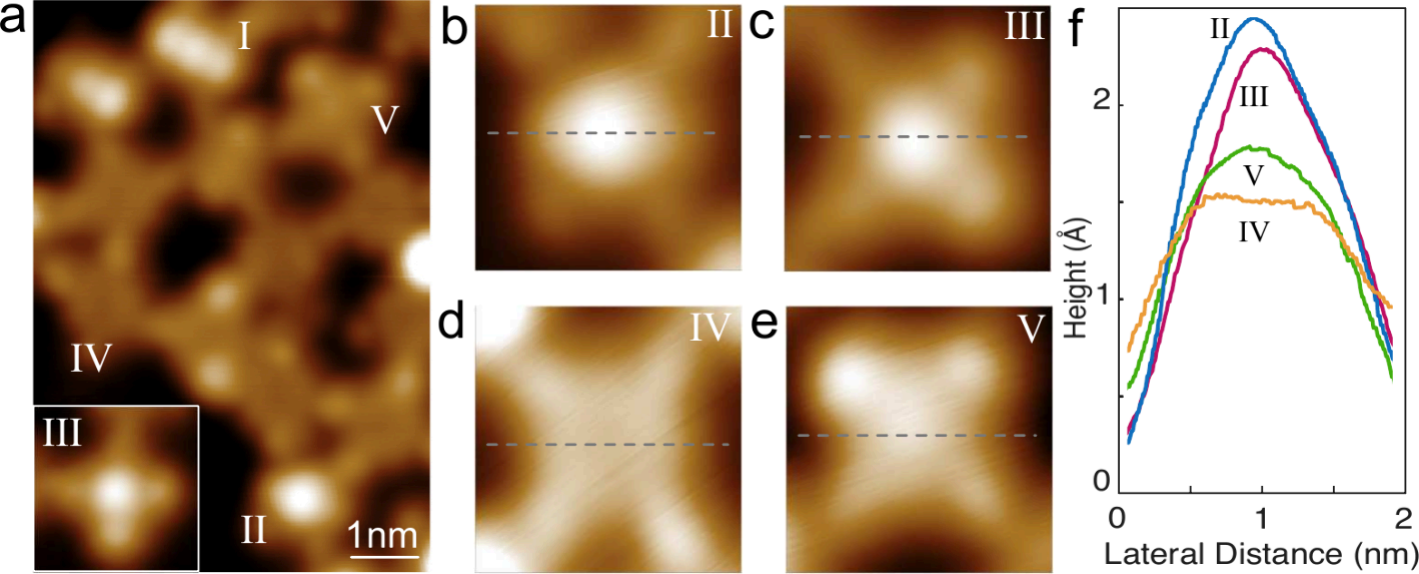
Ullmann coupling reaction on Pb(100). (a) STM image (−60 mV, 10 pA) of a molecular network prepared via Ullmann coupling. Molecules with different topographs and spin states are denoted by I–V. (b–e) Zoom-in topographs (−60 mV, 10 pA) of different species (II–V). (f) Line profiles of different species along the dashed lines in (b–e).

Figure 5 shows the d*I*/d*V* spectra of the five species. Molecule I exhibits a pair of YSR resonances with asymmetry and peak position (−1.36 mV) similar to those of the isolated T-type molecule. For unknown reasons, very few type I molecules exhibit YSR resonances at the gap edge, with asymmetric resonance peaks located outside the gap (Figure S7). YSR excitations are also detected on molecules II and III, but with different peak positions. The Pb coherence peaks are absent on these molecules. On molecule II, the YSR resonances are observed at ±2.06 mV, with significantly higher intensity at negative bias. On molecule III, two pairs of YSR peaks are identified at ±2.11 and ±1.21 mV. Notably, the height asymmetry of the inner pair is reversed compared to the outer pair. This enables us to rule out the possibility that the two pairs originated from the splitting of a single channel due to anisotropy^16,17^ or vibrations,^18^ as these mechanisms result in the same asymmetry for all the YSR peaks. Furthermore, the surrounding molecules do not exhibit any YSR resonances, suggesting that exchange coupling with neighboring molecules^49^ can be disregarded. Therefore, the two pairs of peaks can be ascribed to two distinct YSR channels.

**Figure 5:**
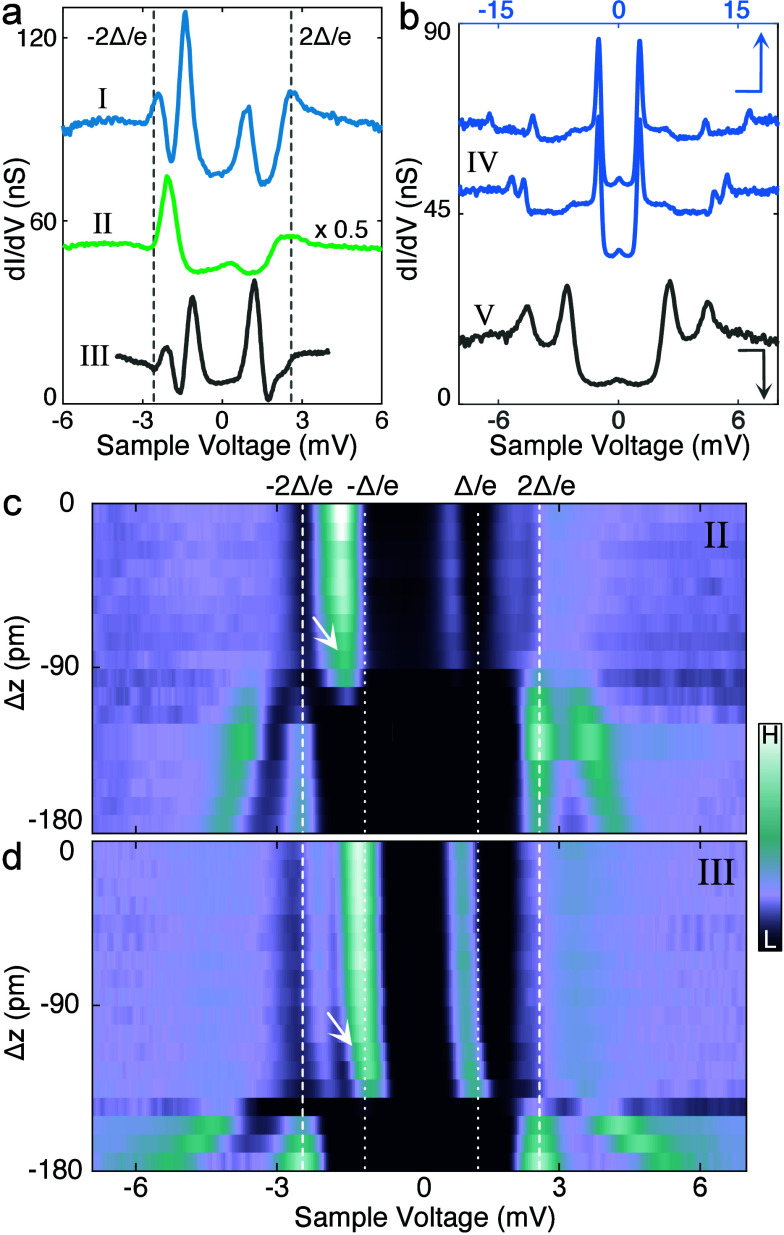
YSR states and spin excitations within the network. (a, b) d*I*/d*V* spectra recorded over the molecules labeled I–V. The tip height was set at −30 mV, 250 pA (a) and −200 mV, 500 pA (b), respectively. (c, d) Color maps of d*I*/d*V* spectra measured at various tip heights on type II (c) and III (d) molecules. The tip height at 0 pm was set at −8 mV, 166 pA (c) and −12 mV, 200 pA (d), respectively. The dashed lines at ±2Δ/*e* mark the superconducting coherence peaks of the surface, and the dotted lines indicate ±Δ/*e*. Thermal replicas of the YSR resonances appear in the latter range.

Similar to the isolated B-type molecule, molecules IV and V exhibit SE accompanied by symmetric coherence peaks (Figure 5b). On molecules IV, two pairs of peaks appear around ±13 mV. The peak positions vary from molecule to molecule, indicating varying axial and transverse anisotropies. After measuring tens of molecules, the ranges of *D* and *E* were found to be 9.8–11.6 and 0.6–2.6 meV, respectively. On molecules V, a pair of SE peaks typically appears much closer to the coherence peaks of the surface. The range of *D* on molecule V is 0.9–3.5 meV. An overview of the parameters relevant for magnetic excitations is presented in Table 2. The values of *D* for molecules I–III are not shown due to the absence of SE resonances, likely due to small anisotropy or short excitation lifetime.^6^ Moreover, renormalization caused by many-body interactions with the surface prevents the independent determination of *D* from the YSR states.^16,17^ After Ullmann coupling, the emergence of multiple YSR resonances and varying magnetic anisotropies can be attributed to strain and molecular deformation caused by the competing effects of intermolecular and molecule–substrate interactions. Such effects also induce distinct electronic characteristics (see the wide range of d*I*/d*V* in Figure S5).

**Table 2:** Summary of the Magnetic Excitation Properties of Various Molecular Species

	I	II	III	IV	V
State	YSR	YSR	YSR	SE	SE
*ρJ*	0.30	0.66	0.71^a^	-	-
			0.32^b^		
*D* (meV)	-	-	-	9.8–11.6	0.9–3.5
*E* (meV)	-	-	-	0.6–2.6	0

a YSR channel 1.

b YSR channel 2.

Molecule I can be converted to molecule IV by slightly reducing the tip height or occasionally during scanning (Figure S8). The subgap YSR states are correspondingly changed to SE peaks.

Similarly, the YSR resonances observed on molecules II and III can be switched to SE by decreasing tip–molecule distance (Figure 5c and d) or lateral manipulation (Figure S9). With reducing tip–molecule distance, attractive force between the tip and molecules increases^6^ and lifts the molecule up, reducing the exchange coupling between the molecule and the surface. As anticipated, a shift of the YSR resonances is observed. For both molecules II and III, the YSR resonances shift toward the Fermi level, indicating the ground (excited) state is a screened (free) spin state (cyan region in Figure 1e). A further decrease in tip height quenches the YSR states and turns SE into the dominant feature in the d*I*/d*V* spectra (Figure 5c and d). The SE peaks are close to the coherence peaks and shift away from the Fermi level as the tip height decreases.

The effective anisotropy energy obtained with varying tip height is 0.94–1.82 meV (II) and 1.88–3.34 meV (III), comparable to those on molecule V. The renormalized anisotropy has been approximately expressed as *D*_*e*_ ≈ *D*_0_[1 – β­(*ρJ*)^2^],^6,50,51^ where *D*_0_ is the bare axial anisotropy, ρ is the normal-state DOS of the substrate at the Fermi level, and β is a constant describing the bandwidth of the exchange interaction. The observed increase in the effective anisotropy (*D*_*e*_) suggests a reduction in the exchange interaction (*J*) and an enhancement of the intrinsic anisotropy term (*D*_0_), similar to the T-to-B transition observed in isolated molecules.

The methodology of on-surface reactions not only expands the structural diversity of molecules but also enriches their magnetic and electronic structures. This versatility provides a degree of control over magnetic impurities, which may turn out useful in the design of spintronic devices.

In summary, we investigated FeTBrPP molecules on a superconducting Pb(100) surface using low-temperature STM, complemented by NRG and DFT calculations. FeTBrPP can adopt various adsorption configurations, both as isolated molecules (T-type and B-type) and within two-dimensional networks formed via Ullmann coupling (five species I–V), each exhibiting distinct magnetic characteristics. By modifying the molecular adsorption site or changing the tip–molecule distance, a transition between YSR states and spin excitations can be induced. This reflects controllable modifications of the parameters governing these magnetic phenomena, the magnetic anisotropy (*D*) and the exchange coupling (*J*). Significant variations in magnetic anisotropy energy (from 0.9 to 11.6 meV) and different quantum phases due to varying *J* were observed across these configurations, underscoring the complex interplay between the molecule and the superconducting substrate.

## Methods

### Synthesis

The FeTBrPP was synthesized according to the procedure described by Y. Li et al.^52^

### Experiment

The measurements were performed with an ultrahigh-vacuum cryogenic STM, at ≈4.6 K (Createc) with base pressures below 1.2 × 10^–10^ mbar. Pb(100) surfaces were cleaned by repeated cycles of Ar^+^ sputtering (1.5 keV) and subsequent annealing to 540 K. The molecules were sublimated from a crucible at 500 K onto a clean Pb(100) surface held at room temperature at pressures ≤ 10^–9^ mbar. Lead tips were cut from a 0.3 mm diameter lead wire, followed by sputtering in a vacuum, and repeated indentation into the substrate. The manipulation of individual molecules was realized at tunneling parameters of 5 mV and 8 nA. Differential conductance spectra were acquired using lock-in detection of the tunneling current by adding an 80 μV_rms_ modulation at 473 Hz to the sample voltage. For spectra covering a wider bias range, a larger modulation of 5 mV_*rms*_ was used.

### DFT Calculations

First-principles calculations were performed using spin-polarized DFT+U as implemented in the SIESTA package.^53–55^ Dispersive interactions were considered via the van der Waals (vdW) density functional by Dion et al.,^56^ reparametrized by Klimeš, Bowler, and Michaelides,^57^ and a mean-field Hubbard correction of *U*_*d*_ = 2 eV for the Fe *d*-orbital.^29^ The Pb(100) surface was constructed by a 4-layer slab, where we adopted an extended double-ζ-polarized (DZP) basis with the orbital radii defined using an energy shift of 100 meV. We used a (35.38 Å)^2^ slab (10 × 10 supercell) for a molecule of size (18.8 Å)^2^ and a cell parameter of 50 Å in the direction perpendicular to the surface to avoid the effects of the interaction between molecules in neighboring cells. The supercell laterally corresponds to a lattice parameter of *a* = 3.53 Å, which is the theoretical value obtained from a bulk relaxation using the same technical details presented here. Due to the dimension of the supercell, if was sufficient to only sample the Γ̅ point of the Brillouin zone to achieve convergence. The integrations in real space were carried out using an energy cutoff of 170 Ry. The core electrons were described by the norm-conserving Troullier–Martins pseudopotentials,^58^ which were generated with the ATOM package included in the SIESTA software. The cutoff radii (all in Bohr) were 1.54 for the *s*, *p*, *d*, and *f* channels of C; 1.84, 2.19, 1.68, and 2.30 for Br; 0.90, 1.36, 1.15, and 2.00 for Fe; 1.48 for every channel in N; 1.98, 2.57, 1.48, and 1.60 for Pb; and 1.25 for H. The molecule and the two Pb surface layers were allowed to relax until a force tolerance of 40 meV/Å was reached. All other slab atoms were fixed at the bulk positions by using the calculated lattice parameter.

### NRG Calculations

NRG calculations were performed using the Ljubljana implementation of the numerical renormalization group (NRG) method.^25^ We used a discretization parameter of Λ = 2 and applied *N*_*z*_ = 32 *z*-averaging to reduce discretization artifacts, retaining up to 12000 states/multiplet. The calculations exploited the SPU(1) symmetry to efficiently handle the spin degrees of freedom. The superconducting gap was set to be Δ = 1.3 meV ( Δ=0.0013D , where the half-bandwidth was taken as D=1 eV). The conduction band was modeled with a constant density of states, ρ=1/(2D) , spanning energies from −D to D . The spin of the impurity was set to *S* = 1.

## Supplementary Material



## Notes

STM topographs and line profiles of molecules within islands and as isolated species, wide range d*I*/d*V* spectra of isolated molecules, d*I*/d*V* spectrum of the T-type molecules under a magnetic field, and STM topographs and d*I*/d*V* spectra of Ullmann-coupled arrays (PDF)
