# Supplementary material for: Magnetic Excitations of Isolated and Interconnected Complexes on a Superconductor
Source: Nano Lett. 2025 Nov 11;25(47):16772–9. doi: 10.1021/acs.nanolett.5c04828 (PMC12670504; doi:10.1021/acs.nanolett.5c04828)
Supplement: Supplementary file 1 [file nl5c04828_si_001.pdf]

Supporting Information for Magnetic Excitations of Isolated and Interconnected Complexes on a Superconductor

Xiangzhi Meng,[*,†] Jenny Möller,[‡] Martin Irizar,[¶,§]

Daniel Sánchez-Portal,[¶,‖] Aran Garcia-Lekue,[¶,⊥] Alexander Weismann,[†]

Rainer Herges,[‡] and Richard Berndt[†]

†*Institut für Experimentelle und Angewandte Physik, Christian-Albrechts-Universität, 24098 Kiel, Germany*

‡*Otto-Diels-Institut für Organische Chemie, Christian-Albrechts-Universität, 24098 Kiel, Germany*

¶*Donostia International Physics Center (DIPC), 20018 Donostia-San Sebastián, Spain*

§*Department of Polymers and Advanced Materials: Physics, Chemistry and Technology, Faculty of Chemistry, University of the Basque Country UPV/EHU, 20018 San Sebastián, Spain*

‖*Centro de Física de Materiales CSIC-UPV/EHU, 20018 Donostia-San Sebastián, Spain*

⊥*Ikerbasque, Basque Foundation for Science, 48013 Bilbao, Spain*

E-mail: meng@physik.uni-kiel.de

Line Profiles and dI/dV Spectra of Further Molecules

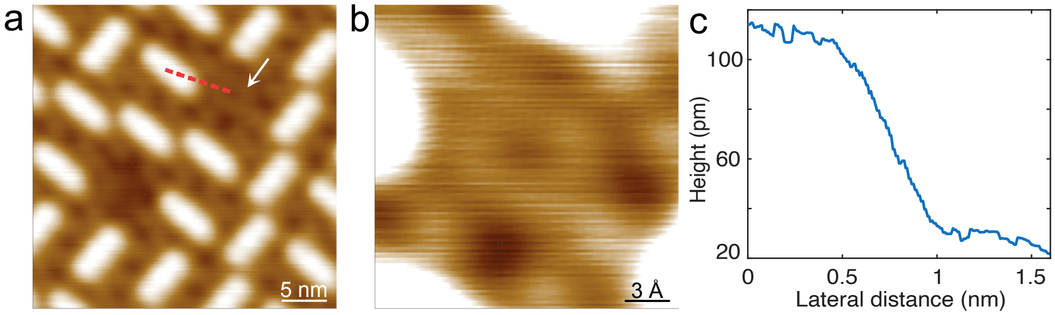

Figure S1: (a) Mono-layer island of FeTBrPP molecules on Pb(100) (−300 mV, 30 pA). (b) Detailed image of a molecule exhibiting spin excitations. The molecule is indicated by the white arrow in (a). (c) Line profile along the red dashed line in (a).

Very few molecules in islands, denoted flat below, do not exhibit a saddle shape (Figure S1 a and b). The apparent height difference between saddle-shaped and the flat molecules measured at the molecular center is approximately 90 pm (Figure S1 c).

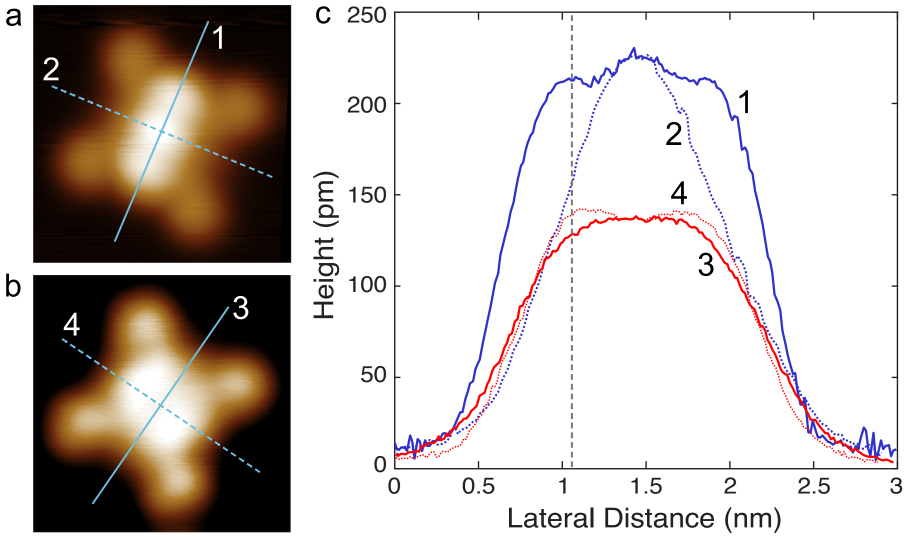

Figure S2: (a) Topograph (−60 mV, 50 pA) of isolated T-type molecule. (b) Topograph (−50 mV, 50 pA) of isolated B-type molecule. (c) Line profiles over the isolated molecules along the lines shown in (a) and (b).

Saddle-shaped and flat molecules in islands appear topographically similar to isolated T-type and B-type molecules (Figure S2a and b). The height difference between T an B is approximately 100 pm (Figure S2c), consistent with the value observed in the layer.

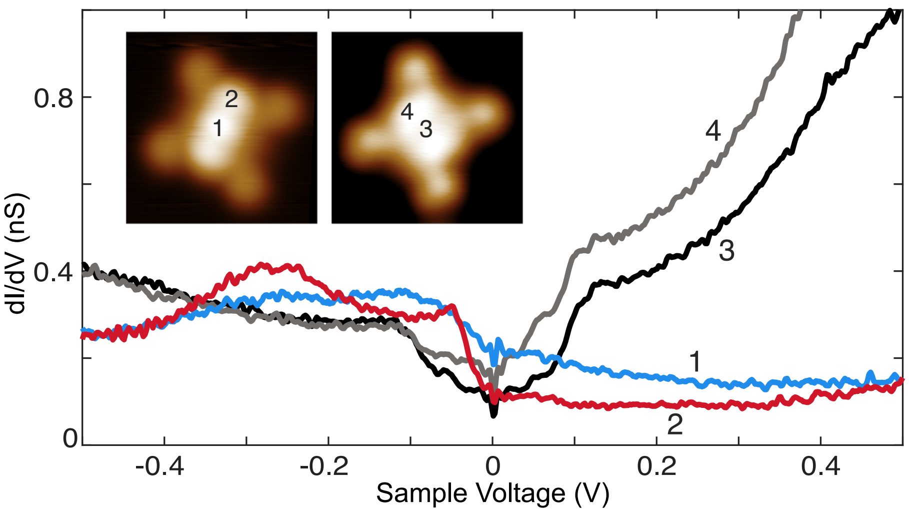

Figure S3: Wide range dI/dV spectrum of isolated T and B molecules. The tip height was set at -0.5 V and 150 pA.

Figure S3 presents dI/dV spectra of isolated T and B molecules. On the T molecule, a broad feature is observed between -0.4 and 0 V. In contrast, the B molecule exhibits a slightly suppressed DOS in this range, accompanied by a step-like structure. At positive bias, the DOS of the B molecule is significantly higher than that of the T molecule. These observations reveal substantial variations in the electronic structure induced by changes in adsorption configuration.

dI/dV Spectrum under Magnetic Field

To estimate the exchange coupling, a magnetic field was applied along the surface normal. At $B = 1$ T, the superconductivity is quenched. In the dI/dV spectrum, a Frota line is observed near the Fermi level instead of YSR excitations, indicating the presence of a Kondo effect. The Kondo temperature T_{K} was determined by matching a Frota function to the data:[1]

$$\rho(\varepsilon) = A_1 \Im[ie^{i\phi}\sqrt{\frac{i\Gamma_{\mathrm{F}}}{\varepsilon - \varepsilon_k + i\Gamma_{\mathrm{F}}}}] + A_2\varepsilon + \rho_0 \tag{1}$$

Here, ϕ is a form factor which determines the line shape and ε_k is the position of the Kondo resonance. The parameter Γ_{F} is related to the half-width at half maximum (HWHM) of the resonance via HWHM $= 2.542\,\Gamma_F$.[2,3] Here, the Kondo temperature is defined as $T_{\mathrm{K}} = \Gamma_{\mathrm{F}}/(1.455\,k_{\mathrm{B}})$,[2] where k_{B} is Boltzmann constant. The fit in Figure S4 yields $T_{\mathrm{K}} \approx 5$ K and $k_{\mathrm{B}}T_{\mathrm{K}} \approx 0.43$ meV $\ll \Delta$, indicating weak exchange coupling between the saddle molecule and the surface.

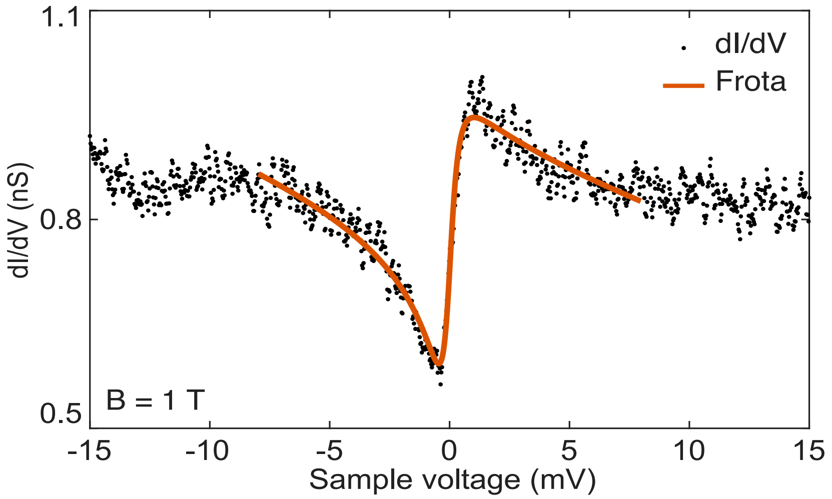

Figure S4: dI/dV spectrum recorded on a saddle molecule under a magnetic field of 1 T oriented along the surface normal. A solid curve shows a Frota function.

Electronic Structures of Interconnected Complexes

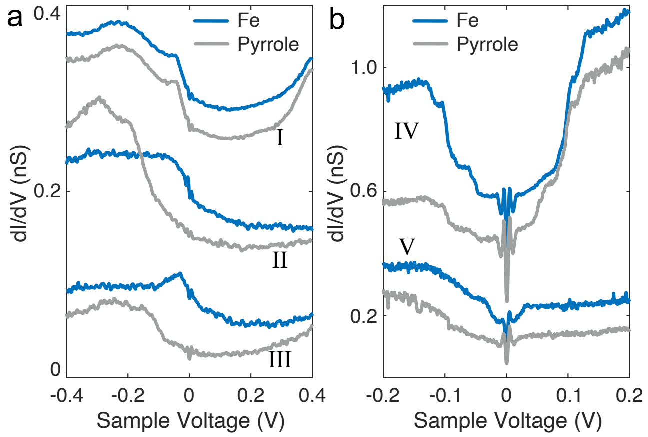

Figure S5: dI/dV spectra over a wide bias range. (a – b) Spectra recorded on the center and the pyrrole groups of the molecules I – V. Set point: (I) −400 mV, 100 pA, (II, III) −500 mV, 100 pA, (IV) −200 mV, 200 pA, and (V) −200 mV, 200 pA.

The different molecular species show distinct electronic characteristics (Figure S5). The electronic structures of molecules I and IV closely resemble those of the isolated T and B molecules, respectively. Among the molecules exhibiting YSR states (Figure S5a), pronounced differences in the DOS are observed on the pyrrole groups: for molecules II and III, the states shift toward lower energies compared to molecule I. Among the molecules with spin excitations (Figure S5b), molecule IV shows the same step features (±59, ±104,and ±128 mV) as the isolated molecule B. On molecule V, only one step is observed at ±27 mV.

STM Overview after Ullmann Coupling

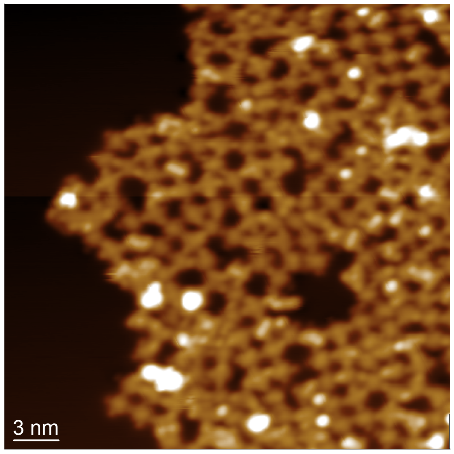

Figure S6: Large-scale STM image (-0.5 V, 20 pA) after Ullmann coupling.

STS on Special Molecules I

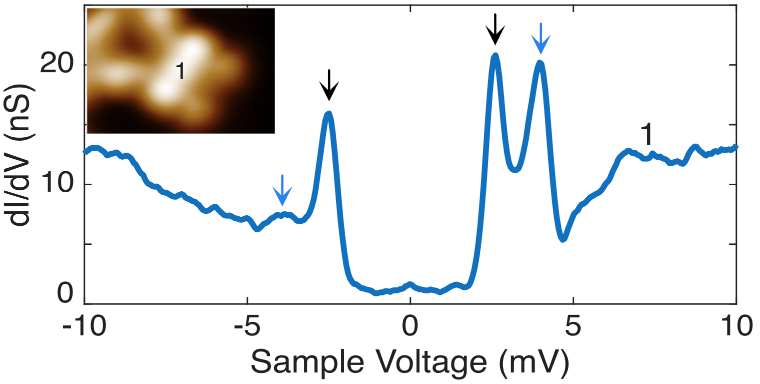

Figure S7: dI/dV recorded on a special molecule I with the tip positioned above the Fe center at a set point of -15 mV and 100 pA. The black arrows indicate the coherence peaks of the superconducting surface. The blue arrows highlight resonances appearing outside the superconducting gap.

Very few molecules I exhibit distinct spectra (Figure S7). The coherence peaks (black arrows) show noticeable asymmetry, which may arise from weak exchange coupling with the surface. Unlike symmetric SE resonances, a pair of asymmetric peaks appear outside the superconducting gap, whose origin is presently unclear.

Switching During Scanning

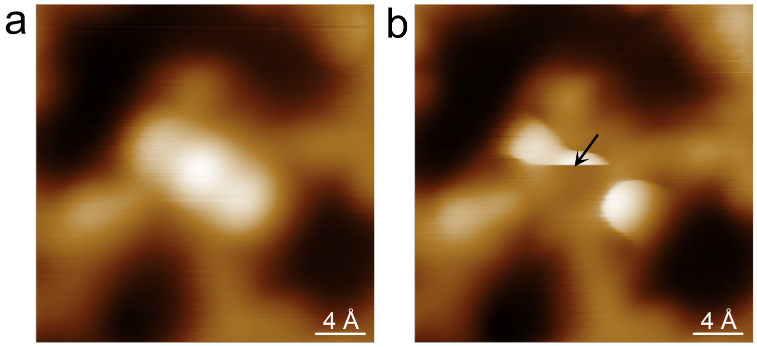

Figure S8: (a) Topograph (−6 mV, 10 pA) of a molecule in a network. (b) The same molecule imaged at reduced tip-molecule distance (−6 mV, 100 pA).

The structure of saddle-shaped molecules can be easily affected by the STM tip, especially when the tip-molecule distance is reduced (black arrow in Figure S8b).

Lateral Manipulation of a Molecule after Ullmann Coupling

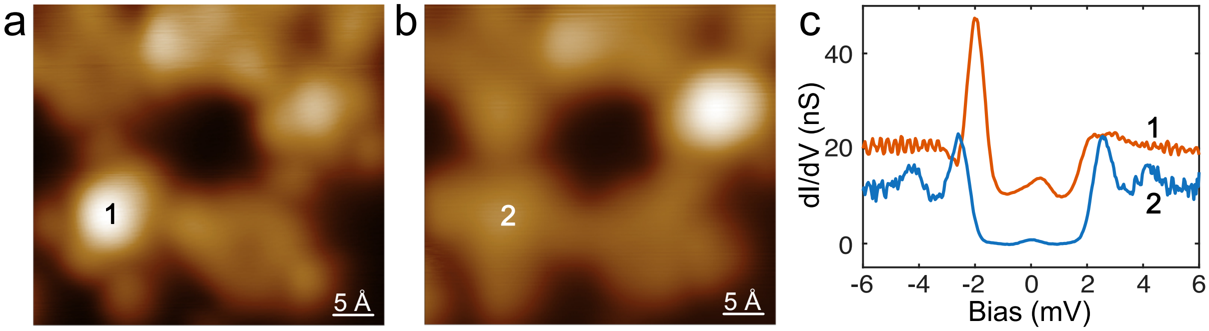

Figure S9: (a) and (b), topographs (−60 mV, 10 pA) recorded before and after manipulation. (c) dI/dV spectra obtained at the positions denoted by (a) 1 and (b) 2.

Cluster of a few molecules at an edge of a coupled two-dimensional network can be manipulated with the STM tip by pushing laterally. Figure S9a and b shows an example of topographs before and after such a manipulation. The molecule in the left bottom corner is switched between type II and V as classified in Figure 4. Correspondingly, YSR excitation and SE are detected on this molecule before and after the manipulation, respectively, as shown in Figure S9c.

References

1. Prüser, H.; Wenderoth, M.; Weismann, A.; Ulbrich, R. G. Mapping Itinerant Electrons around Kondo Impurities. *Phys. Rev. Lett.* **2012**, *108*, 166604.

2. Prüser, H.; Wenderoth, M.; Dargel, P. E.; Weismann, A.; Peters, R.; Pruschke, T.; Ulbrich, R. G. Long-Range Kondo Signature of a Single Magnetic Impurity. *Nat. Phys.* **2011**, *7*, 203–206.

3. Gruber, M.; Weismann, A.; Berndt, R. The Kondo Resonance Line Shape in Scanning Tunnelling Spectroscopy: Instrumental Aspects. *J. Phys. Condens. Matter* **2018**, *30*, 424001.
